# Review of Current Human Genome-Scale Metabolic Models for Brain Cancer and Neurodegenerative Diseases

**DOI:** 10.3390/cells11162486

**Published:** 2022-08-10

**Authors:** Ali Kishk, Maria Pires Pacheco, Tony Heurtaux, Lasse Sinkkonen, Jun Pang, Sabrina Fritah, Simone P. Niclou, Thomas Sauter

**Affiliations:** 1 Department of Life Sciences and Medicine, University of Luxembourg, L-4367 Belvaux, Luxembourg; 2 Luxembourg Center of Neuropathology, L-3555 Dudelange, Luxembourg; 3 Department of Computer Science, University of Luxembourg, L-4364 Esch-sur-Alzette, Luxembourg; 4 NORLUX Neuro-Oncology Laboratory, Luxembourg Institute of Health, Department of Cancer Research, L-1526 Luxembourg, Luxembourg

**Keywords:** brain metabolism, metabolic modelling, glioma, neurodegenerative diseases, astrocyte, neuron

## Abstract

Brain disorders represent 32% of the global disease burden, with 169 million Europeans affected. Constraint-based metabolic modelling and other approaches have been applied to predict new treatments for these and other diseases. Many recent studies focused on enhancing, among others, drug predictions by generating generic metabolic models of brain cells and on the contextualisation of the genome-scale metabolic models with expression data. Experimental flux rates were primarily used to constrain or validate the model inputs. Bi-cellular models were reconstructed to study the interaction between different cell types. This review highlights the evolution of genome-scale models for neurodegenerative diseases and glioma. We discuss the advantages and drawbacks of each approach and propose improvements, such as building bi-cellular models, tailoring the biomass formulations for glioma and refinement of the cerebrospinal fluid composition.

## 1. Introduction

In Europe, 169 new million cases of brain disorders were reported in 2019 [1]. Neurological disorders, brain and central nervous system (CNS) cancer, strokes, and mental disorders are all examples of brain disorders [2]. The high toll on the life quality of patients suffering from neurodegenerative diseases (NDD) and the societal burden that are increasing with the ageing of the western population. Alongside cardiovascular diseases and cancer, NDD are a major health care challenge, with dementia being the most expensive disease to manage [3]. While the annual cost of dementia is 1.5 times more than cancer in the UK, research funding for dementia is only 30% of cancer [4]. Brain cancers can be considered rare diseases with an estimated 308,000 new cases and 251,000 new deaths worldwide in 2020 [5] of which glioblastoma (GBM) accounts for more than half of malignant CNS cancers [6]. However, unlike NDD, which develops over decades, the life expectancy of GBM patients is 5% survival over five years [7]. However, both NDD and GBM are incurable, age-related (the median age of diagnosis for GBM is 65 years old [6]), and show metabolic deficiencies or rewiring that could be exploited as potential drug targets [8].

**Figure 1:**
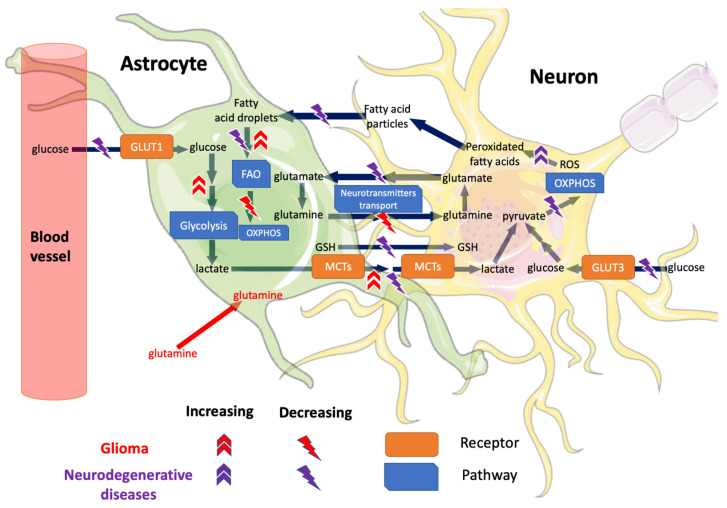
Dysregulated metabolic reactions between astrocytes and neurons in healthy conditions, NDD and glioma. Under healthy conditions, astrocytes provide metabolic support with nutrients to neurons and carry out neurotransmitter and ROS detoxification [25]. As glial cells are becoming malignant in glioma, they shift from OXPHOS to glycolysis [16] and FAO [26] for energy generation. Moreover, astrocytic glutamine transport to the neuron is disrupted [27] in glioma, and glutamine uptake by the glial cell is increased [12]. Meanwhile, in NDD, neurons shift to reduced glycolysis and OXPHOS to decrease the produced energy [25]. In some NDD, the bi-cellular transport from astrocytes to neurons of both GSH and glutamate are decreased [25], with the former accumulating ROS and peroxidated fatty acids from the neuronal activity [28]. The peroxidated fatty acids are exacerbated by the deceased astrocytic FAO. Because of the difference in astrocytic glycolysis between glioma and NDD, astrocytic lactate transport to the neuron is increased in glioma [29]; meanwhile, it is decreased in NDD [25]. Other cellular interactions were excluded for simplification, such as astrocyte–glioma cell interactions [30], oligodendrocytes, microglia and the different neuron cell types. FAO: fatty acid oxidation, GLUT1/3: glucose transporter 1/3, GSH: glutathione, MCT: monocarboxylate transporters, OXPHOS: oxidative phosphorylation, ROS: reactive oxygen species. Parts of the figure were drawn by using pictures from Servier Medical Art. Servier Medical Art by Servier is licensed under a Creative Commons Attribution 3.0 Unported License (https://creativecommons.org/licenses/by/3.0/).

Lower grade gliomas (LGG), a less aggressive glioma form than GBM, is more heterogeneous in prognosis and response to treatment and is characterised by lower proliferation speed [9]. More than 80% of LGG have mutations in isocitrate dehydrogenases that play a central role in metabolism as catalysing reaction in the Krebs cycle, redox homeostasis, biosynthesis of lipids, and glutamine metabolism [9,10]. These LGG are classified into astrocytoma (AST) and oligodendroglioma (ODG) based on the glial cell type they originated from. GBM and LGG also have different preferred energy sources. Glucose, the main fuel of neurons [11], and glutamine, required for the biosynthesis of neurotransmitters [12], are abundant in the brain microenvironment and have been linked to GBM invasion [13,14]. The Warburg effect is a hallmark of GBM with a shift from oxidative phosphorylation (OXPHOS) and TCA cycle to glycolysis for energy production [15]. The upregulation of glycolysis and downregulation of OXPHOS and TCA is linked to poor survival in GBM [16]. This increased glycolysis rate, even under hyperoxia, increases GBM chemoresistance [17]. Furthermore, this metabolic shift allows channelling carbon and nitrogen fluxes into the biosynthesis of nucleotides via the pentose phosphate pathways (PPP) [18]. The PPP also permits reducing NADP+ to NADPH and hence maintains oxidative homeostasis [18]. A lesser-known GBM subtype, mitochondrial GBM, was identified by multi-omics analysis with decreased glycolysis and increased OXPHOS (reverse Warburg effect) [19]. This reverse Warburg effect occurs in late tumour formation and is characterised by sensitivity to OXPHOS inhibitors [19]. Neuron-glioma metabolic interactions through neurotransmitters can change glioma progression [20]. Mainly, dysregulation in neurotransmitter exchange such as of glutamine and gamma-aminobutyric acid (GABA) emerges as part of the GBM metabolic remodelling [20]. Glutamine, a neurotransmitter precursor, is required in glycolytic cells to fuel the TCA cycle and the biosynthesis pathways. Unlike GBM, LGG shows low glycolysis [21], which may explain their relative decreased proliferation and ODG growth is robust to glutamine starvation [22]. Similarly, GABA may be linked to increased GBM stemness [23] where the pharmacological inhibition of GABA release of the GBM cells reduced GBM growth [24]. The main astrocyte-neuron metabolic interactions under healthy conditions in addition to glioma and NDD are summarised in Figure 1.

Besides glycolysis and neurotransmitter metabolism, deregulation of the lipid metabolism, notably cholesterol metabolism, was shown to accumulate in GBM due to an increase in uptake and a downregulation of the efflux pathway. Cholesterol accumulation is a hallmark of cancer [31]. Due to the role of cholesterol in signalling and membrane plasticity, deregulation of cholesterol pathways often leads to uncontrolled proliferation and cell invasion and migration. Cholesterol can scarcely pass through the brain–blood barrier (BBB) [32]. This limits the pool of cholesterol in the brain, which is synthesised mainly by the astrocytes. The effect of cholesterol biosynthesis on GBM is debated, and the results are not consistent between studies. Cholesterol biosynthesis was found upregulated in GBM neurospheres, tumour initiating cells, cell lines, and patient samples, and a factor related to decreased patient survival and tumour growth [33,34,35,36]. This upregulation allowed statins (cholesterol biosynthesis inhibitor) to reduce the growth of GBM tumour initiating cells [35]. In another study, cholesterol biosynthesis has reduced expression in GBM cell lines [37]. The reduced cholesterol biosynthesis in GBM cell lines is supported by GBM resistance to statins [38]. On the other hand, AST cells with an upregulation in this pathway are sensitive to atorvastatin [39]. Besides cholesterol deregulation, an upregulation of fatty acid synthesis, and beta-oxidation has been described in GBM [26]. In a nutrient-rich microenvironment, beta-oxidation channels fatty acids to cancer cell proliferation. Meanwhile, in lower nutrient levels, fatty acids are diverted to OXPHOS to produce ATP and precursors for amino acids and nucleotide synthesis. Inhibition of fatty acid oxidation (FAO) and carnitine transport show synergistic effects in GBM cell lines’ survival [40]. Moreover, the transporter of very long fatty acids SLC27A3 is upregulated in glioma but not in the healthy brain and are linked to patient survival. Genetic knockout of SLC27A3 decreased stearic acid uptake and reduced the GBM cell line U87MG growth [41]. Recently, some GBM xenografts were found to be resistant to glycolysis inhibitors with upregulation of OXPHOS and dependency on FAO [42]. Combination of glycolysis and FAO inhibitors synergistically decreased the growth of these resistant xenografts [42]. These studies show GBM’s ability to shift energy dependency from glycolysis to FAO and the potential of FAO pathway that could be exploited for drug repurposing [41].

Besides neurons and astrocytes that play a central role in gliomas, other glial cell types, oligodendrocytes and microglia, were described to play a role in tumour progression [43]. Oligodendrocytes are cells engulfing the neuron axon with the myelin sheath to maintain neuronal signal [44]. Similar to astrocytes, oligodendrocytes provide metabolic support of nutrients to neurons such as lactate and pyruvate [44]. In addition to ODG and mixed glioma originating from oligodendrocytes, oligodendrocytes increase the invasiveness of GBM [45]. Microglial cells are the resident immune cells of the CNS, dedicated to the maintenance of CNS homeostasis. These cells are implicated in numerous processes essential for tissue development and maintenance–remodelling–repair of the neural environment [46]. Microglia play important roles in the adult brain but also earlier during brain development [47]. These cells are able to eliminate extra synapses (synaptic pruning) but also to eliminate dying neurons by phagocytosis. Microglia are also devoted to rapidly reacting to any kind of pathological insults (pathogens, debris, dying cells, aberrant proteins) [48]. Similar to macrophages, microglia generate an immune response to pathogens or any insults [49]. An excessive microglial reactivity can play a critical role in the development and progression of brain diseases.

Microglia can switch from a quiescent state to pro-inflammatory or anti-inflammatory phenotypes and vice-versa [50]. This change of phenotype is often accompanied by metabolic shifts [51]. Pro-inflammatory microglia are known to quickly release a large panel of pro-inflammatory compounds such as cytokines, chemokines but also reactive oxygen and nitrogen species (ROS/RNS) [51]. Anti-inflammatory microglia will be important in order to calm down the inflammation and to favour tissue repair [51]. For this purpose, anti-inflammatory microglia produce high levels of anti-inflammatory cytokines. The expression of anti-inflammatory phenotype biomarkers can be used to differentiate between grade 2 and 4 astrocytoma [52]. In addition, GBM subtypes show significant percentages of microglia cells in the microenvironment, with the mesenchymal subtype having the highest percentage and lowest survival [53]. Microglia, monocytes, and macrophages make nearly 30–50% of the GBM tumour weight [54]. Little is known about the exact metabolic role of the two microglia phenotypes in GBM [29]. While both phenotypes are expressed in the different stages of GBM, more pro-inflammatory microglia are activated in early glioma development using glycolysis and OXPHOS for energy [29]. In a second stage, the pro-inflammatory microglia depend on glycolysis mainly due to inflammation-induced hypoxia. This second stage is characterised by nitric oxide formation and lactate production [29]. Lastly, the high concentration of lactate in the microenvironment and lack of oxygen favour the anti-inflammatory phenotype. The overrepresentation of anti-inflammatory macrophages in glioma induces immunosuppression, increasing glutamine uptake and angiogenesis through vascular endothelial growth factor (VEGF) expression [29].

Despite the diversity of NDD pathologies, including Parkinson’s (PD), Alzheimer’s (AD), Huntington’s and amyotrophic lateral sclerosis, they share several metabolic hallmarks. Cell death of neurons in many NDD has been observed due to protein misfolding and accumulation [55]. Ageing, oxidative stress, and mutations are the main factors for protein misfolding [56]. The pathological protein accumulation can be either intra- or extracellular depending on the disease [57]. This in turn causes malfunctions with membrane receptors and further distribution in the neural signalling [57]. Moreover, protein accumulation increases lipid oxidation and mitochondrial dysfunction [28]. Glial cells such as astrocytes and oligodendrocytes show a supportive rule in alleviating the cellular damage and redistributing metabolites to neurons in NDD [58]. Similar to neurons, cellular damage in astrocytes and oligodendrocytes occurs due to protein accumulation that causes loss of normal functions such as the distribution of neuronal lactate uptake from glial cells and gain of toxic functions [59,60]. Hypomyelination of oligodendrocytes induced by protein accumulation is further accelerating neuron damage [60]. Microglia protect from neurodegeneration by maintaining synaptic remodelling and phagocytosis of dead cells. Similar to neuron cells, intracellular protein accumulation may cause loss of the astrocytes and microglia normal functions that may aggravate NDD [59,61]. An increase in microglial phagocytic activity has been shown concomitant to an increase in the production of anti-inflammatory mediators and a decrease of pro-inflammatory mediators [62]. Balance between pro-inflammatory/anti-inflammatory microglia activation shows improved prognosis and treatment of NDD [63]. Mainly, shifting from pro-inflammatory to anti-inflammatory activation decreased neuroinflammation in some NDD [64].

In most NDD, neuronal glucose uptake is downregulated and glucose metabolism is impaired [65]. The alteration in glucose metabolism and the downregulation of GLUT transporters lead, together with the mitochondria dysfunction, to lower energy levels that aggravate the pathologies. Mitochondrial dysfunction does not only impair cellular energy, but as mitochondria play a key role in calcium and redox homeostasis, they also contribute to redox stress. Furthermore, dysfunction in OXPHOS increases the production of ROS that will further increase mitochondrial damage and eventually initiate apoptosis [66]. Lipid peroxidation is another hallmark of many NDDs in early development due to mitochondrial damage and increased ROS [28]. Some by-product metabolites of lipid peroxidation are potential biomarkers for different NDDs such as isoprostanes in AD and malondialdehyde in PD [66]. Lastly, metabolism of polyamines such as spermidine and spermine is also deregulated in NDD. Both metabolites are antioxidants and have antiapoptotic properties with expression in neurons and glial cells. Deregulated polyamine metabolism was detected in AD, and PD and was accompanied with mitochondrial damage and apoptosis [67].

Constraint-based metabolic modelling (CBM) and genome-scale metabolic models (GEM) are commonly applied to study metabolism and, notably in cancer, where it was used to understand rewiring strategies and predict repurposable drugs [68] and drug off-targets [69]. GEM is an in silico representation of the metabolism where the interactions between metabolites and the biochemical reactions are formulated in a sparse stoichiometric matrix and the relationship between genes and reactions by Boolean rules (GPR rules). Moreover, GEM is used to simulate the role of the microbiome in the development of PD [70,71] or to study psychiatric diseases [72] and AD [73] in humans and PD-like phenotypes in mice [74]. However, brain metabolism has specific properties that must be considered before applying CBM. The brain is protected by the BBB that controls the exchange of metabolites between cerebrospinal fluid (CSF) and the blood [75]. The permeability of the BBB can be altered in numerous diseases, which also impacts the CSF composition and the brain microenvironment [76], a feature that can be further used to constrain metabolic models. Furthermore, the metabolism of neurons and glial cells is interconnected and numerous exchanges between glial cells, notably astrocytes that are part of the BBB, have been described. For instance, glial cells store glucose in the form of glycogen, and, when required, glycogen fuels glycolysis [77]. The produced lactate can then be taken up by surrounding neurons [78]. Hence, for the study of some diseases, a bi-cellular or multicellular model is more suitable than an averaged brain model that lacks the required resolution.

In this review paper, we survey brain GEMs that could be used to study brain cancer, NDD or other brain disorders. We focus mainly on the modelled cell type, the type of model (single versus bi-cellular models), the curation level and the overall quality of the model in terms of the gene, metabolite and reaction annotations. We further consider the type and quality of data used to support the inclusion of reactions in the models as well as the validation used in the different studies. The model size, the inclusion of cell type-specific pathways and the optimisation function were also used to assess the models’ completeness and specificity. We further compare the metabolite composition in models with a biomass function to assess their specificity in the investigated system. Finally, we highlight the strengths of the different GEMs, in terms of applied constraints, data utilised for model-building and validation that could be incorporated in future models and suggest some improvements.

## 2. Materials and Methods

### 2.1. Literature Search for Manually Curated Brain GEMs

An extensive literature review was performed to gather brain GEMs. To distinguish between the different curation levels, we classified the metabolic models into three classes: curated, semi-curated, and automatically generated (AG). In this review, we focused mostly on curated and semi-curated models.

Curated: models built starting from a list of biochemical reactions collected from literature or databases to which reactions were then added to fill the gaps or add the missing information. Alternatively, the starting point can also be an automatically generated GEM. However, most pathways have been carefully checked to eliminate reactions with no or low support from the literature that is not required for modelling purposes and to add missing reactions but are known to be present in the studied system.

Semi-curated: models built using a generic reconstruction or an automatically generated model that was curated via the addition of constraints, modifications (addition and removal of reactions) in key pathways or required to combine two models in a bi-cellular model.

Automatically generated: models built automatically using a model-building algorithm such as FASTCORE [79], FASTCORMICS [80], rFASTCORMICS [68], GIMME [81], mCADRE [82], PRIME [83], iMAT [84], RegrEX [85], and tINIT [86] from a GEM, and expression data (transcriptomic or proteomic) without or with limited manual curation.

### 2.2. Inclusion and Exclusion Criteria of Publications

Publications focusing on building curated and semi-curated GEMs for the human brain and using CBM were included. In addition to curated and semi-curated models, only one AG GEM was included for relevance to GBM. Five types of brain GEMs were excluded from this review: (1) AG GEM without validation, (2) curated GEM with a follow-up included in the present review, (3) dynamic metabolic models in the brain, (4) publications with no publicly available model files in the supplementary files or BioModels, and (5) Non-human GEM. Dynamic metabolic models were excluded as they are out of the scope of the current review, and they were already covered in a previous review [87]. We also focused on human GEM as being more relevant for personalised medicine. Due to missing abbreviated names for some GEM, we referred to each model by the last name of the first author and the date of the publication.

### 2.3. Metadata Gathering for Determining the Extensiveness of the Manual Curation

After selecting brain GEM publications, basic information was retrieved from each publication regarding the model used as template, cell type, diseases, and data used during model building or validation. Moreover, the detailed types of the different omics and experimental data were collected with the number of samples to identify the extensiveness of the manual curation of the model.

### 2.4. Determining Model Sizes and Common Genes

The model files were imported using the COBRA Toolbox V3 [88], and the number of reactions, genes and metabolites were determined. The median, minimum and maximum numbers were computed for publications with more than two GEM. Since some reactions may not be able to carry a flux at all, the number of flux-consistent reactions were identified using FASTCC [79]. Moreover, the brain GEMs’ model genes were mapped to ENTREZ IDs to compare the overlap between the different models using the UpSet plot in R. Two generic models, Recon3D and Human1, were used in the gene overlap analysis. The intersection and the union of the model genes were retrieved for publications with more than two GEM.

### 2.5. Determining the Level of Completeness and Specificity of the Brain GEM

To evaluate the specificity and the completeness of the human brain GEM, tissue gene categories were retrieved from the Brain Atlas [89] of the Human Protein Atlas (HPA) [90]. HPA classifies the protein-coding genes into five categories according to the expression level in a target tissue compared to other tissues. These categories were retrieved for the human brain and mapped to the ENTREZ identifiers. The five categories were grouped for simplification into two types: Supported (which includes “Elevated in brain”, “Elevated in other but expressed in brain” and “Low tissue specificity but expressed in brain”), and unsupported (which includes “Not detected in brain” and “Not detected in any tissue”). The different HPA data were mapped to the genes of the brain GEM, and to two generic models, Recon3D and Human1, to assess the fraction of genes of each model that are supported or unsupported in the brain by the HPA protein data. Two scores were calculated for each brain GEM, specificity and completeness. Model specificity (indicated as two numbers) is the number of supported or unsupported genes in each GEM. In contrast, model completeness is the ratio of the model supported or unsupported genes to the total count of genes in each category.

### 2.6. Evaluation of Objective Function and Validation Used in the Brain GEM

The brain GEMs were further evaluated by their objective function (OF) and used validation data to determine the strengths and limitations of these GEM. Different brain GEMs include different OFs depending on the diseases of interest. These OFs were categorised into tailored or generic based on the manual curation. In addition, the rationale for choosing a specific OF according to the research question was summarised. The OFs of the GBM GEMs were compared using their metabolite composition. Moreover, the data used for validation in the brain GEMs were outlined and their importance for the research question was discussed. Finally, the limitations and the strengths of the brain GEMs were summarised based on the choice of the template model, model-building technique, study design, use of constraints data, presence of sink reactions, heuristic thresholds in the discretisation of the expression data, and applying standard identifiers.

## 3. Results

In this review, we discuss nine publications that focus on reconstructing GEMs that could be employed for NDD, brain cancer and other brain diseases. The selection of these models was based on their public availability (see Supplementary File S1 Table S1), the curation level and/or the pertinence to GBM. By curation, we understand the contextualisation of the models with constraints retrieved from literature or published experimental data, the addition of reactions specific to the cell type of interest, the choice of the OF and if the OF was tailored to the cell type of interest. Finally, we discussed the validations used in the different publications and the strengths and limitations of these GEMs.

### 3.1. Selected Brain Metabolic Models Could Be Potentially Reused for NDD and Glioma

The main difference between the curated and semi-curated is the extension of the curation. For example, Thiele2020, considered curated, defines 578 core reactions (reactions supported by literature in the brain) and added 43 metabolites to the list of metabolites passing BBB. While Baloni2020 completed the list of BBB metabolites with an additional 372, no core reactions were added. Five curated, three semi-curated and one AG GBM GEM were selected. Most of these GEMs integrated transcriptomic and proteomic data for model-building, while only two GEMs used metabolomics data to define exchange reactions (see Supplementary File S1 Table S2).

### 3.2. Lewis2010 (iNL403)

Lewis2010 [91] is a bi-cellular GEM of a neuron and an astrocyte with 1073 reactions and 987 metabolites [91]. This GEM was built by extracting the reactions of glycolytic, mitochondrial, and transport pathways from the generic reconstruction Recon 1 [92]. The presence of each of these reactions in the brain was determined based on expression from different sources. Lewis2010 was curated by adding brain cell type-specific (astrocyte and neuron) biochemical reactions [91]. The models were then contextualised using manually selected neuron cell type-specific reactions to build glutamatergic, GABAergic, and cholinergic neurons. In addition, the model bounds were constrained using uptake rates obtained from the literature.

### 3.3. Sertbaş2014 (iMS570) from Tunahan Çakır Lab

Sertbaş et al., 2014 [93] expanded the brain reconstruction of Çakιr et al., 2007 to obtain a bi-cellular astrocyte and neuron model with 630 reactions and 530 metabolites from the literature [93]. ATP production and glutamine/glutamate exchange were added as OF, whereas GABA exchange was included to ensure the coupling of the exchange reactions between the astrocyte and the neuron model.

### 3.4. Özcan2016 (iMS570g) from Tunahan Çakır Lab 

Since the curated Sertbaş2014 only includes non-cancerous OFs, Özcan et al., 2016 [94] added 29 reactions to integrate a tailored growth OF [94]. Among the 29 reactions, 25 are biomass-related and four reactions are linked to glutamine metabolism in GBM. The tailored OF was formulated based on the contribution of both the astrocyte and the neuron to the dry weight of the white matter.

### 3.5. MartínJiménez2017

A curated astrocyte GEM [95] was reconstructed using the Human Metabolic Atlas (HMA) [96] and microarray data of foetal cortical astrocytes. The completeness of the model was assessed by identifying gaps that were filled by adding astrocyte-specific reactions based on enzymes present in the HPA [90]. Lastly, experimental constraints specific to hypoxia were used to compare the activated reactions under normal and hypoxic conditions.

### 3.6. Thiele2020 

Thiele et al., 2020 [97], built two sex-specific multi-tissue models (Harvey and Harvetta for male and female, respectively) of 26 organs with >80,000 reactions [97]. Reactions for the protein and drug metabolism pathways were removed initially from the Recon3D model [98], before assembling according to the connections of the different organs. The two multi-tissue models were built using FASTCORE [79] from the assembled reconstructions and organ-specific core reactions from omics data and literature. Exchange reactions of the organs and extracellular fluid such as the CSF were constrained by metabolomics data from the Human Metabolome Database [99]. Meanwhile, the exchange reactions between the extracellular fluid of the different organs and the systemic blood circulation were obtained from the literature. Moreover, organ-specific models were further extracted from the two multi-tissue models as standalone consistent GEMs. The man and woman brain GEMs will be referred to as Thiele2020_Harvey and Thiele2020_Harvetta.

### 3.7. Baloni2020 

Baloni et al., 2020 [100] built seven brain region-specific GEMs using the Recon3D model [98] and transcriptomic data from different brain regions of healthy and AD patients with the mCADRE algorithm [82]. The reactions of the drug metabolism pathway were removed from the Recon3D model. Then, the transcriptomic data were discretized using the top 25th percentile cut-off to obtain a set of reactions used as input for mCADRE. After the building, the model was constrained using metabolites passing the BBB from Thiele2020, bile acid metabolites from targeted metabolomics of brain samples, uptake rates obtained from Lewis2010 and other literature sources were integrated. Furthermore, gap filling was performed using HPA expression [90] to determine gene presence. Finally, the OF of Sertbas2017 was integrated into the GEM.

### 3.8. EcheverriPeña2021 (Neuro-Glia_GEM) 

EcheverriPeña et al., 2021 [101] integrated two AG GEMs [102], to build a bi-cellular neuron-glia metabolic model. These models were obtained using Recon 2 [102] and HPA [90] as input for the MinMax algorithm [103]. To identify the metabolic pathways changes related to Arylsulphatase A (ARSA) deficiency, EcheverriPeña et al., 2021 added reactions of sulfatide degradation from the myelin band. The added reactions made the glial cellular compartment more specific for oligodendrocytes.

### 3.9. Lam2021

Lam et al. [104] analysed telomeric ageing in AD and PD compared to healthy controls by aggregating gene expression data from six sources via batch correction. The combined AD and PD samples were stratified into three subclasses using unsupervised clustering. Four semi-curated GEMs were built from the expression of the three clusters in addition to the control samples using tINIT [86] from the RAVEN Toolbox [105]. The template model for model-building was an adipocyte GEM, iAdipocytes1850 [106], after mapping the gprRules from the generic reconstruction HMR3 [107] and constraints from Baloni2020 [100]. Flux balance analysis and reporter metabolite analysis were applied to define the different pathways and metabolites between the three combined AD-PD GEMs and the control GEM. These pathways and metabolites were validated using semi-curated Zebrafish GEMs built from normal and enhanced ageing. The Zebrafish GEMs were built from Zebrafish expression data of wildtype and mutant TERT gene responsible for telomere maintenance.

### 3.10. Larsson2020 [108]

Larsson2020 [108] merged 139 patient-derived AG GEMS to build a GBM model using tINIT [86]. These 139 AG GEMs were built by Uhlén et al. [109] using the generic reconstruction HMR2 [107] and the RNA-Seq data of GBM from the TCGA-GBM dataset [110]. Furthermore, single-gene deletion was performed on both the patients and the generic GBM models using FastGeneSL [111]. Then, the genes whose in silico knockout might affect healthy tissues were excluded by evaluating the effect of a knock-out on 77 pre-defined metabolic tasks (defined as metabolites that must be produced from a defined minimal media or a set of metabolites) on an AG healthy brain model from the HMA [96]. The different data used by the brain GEMs, their curation status and cell types are summarised in Table 1.

### 3.11. Manual Curation Included Tissue-Specific Constraints, Added Reactions, and Compartments

A curation can either be a refinement of a curated or an AG GEM by the addition or removal of reactions, metabolites and flux rates. Four models incorporated experimental flux rates to contextualise their models to represent healthy brain cell models (Sertbaş2014, Lewis2010 and MartínJiménez2017), and GBM (Özcan2016). Most experimental flux rates are specific to a cell type (mostly glial or neuronal), while others, such as glucose uptake, are measured at the BBB. While Sertbaş2014 assumed equal glucose consumption for the glial and neuron model, Özcan2016 divided the overall brain glucose, oxygen and glutamine uptakes based on the neuron and glial proportion in the white matter mass.

Four models included a compartment to simulate the exchange between the models and the BBB, Lewis2010, MartínJiménez2017, Thiele2020, and Baloni2020. Furthermore, metabolites that cannot cross the BBB were defined in Thiele2020, and the respective transporters were removed. Overall, to better model the physiology of the studied diseases, the models have to be adapted by adding or removing reactions or by applying the constraints based on experimental measurements obtained from diseased patients or cell lines. Besides whether there is binary information if a metabolite passes or not passes the BBB, or what metabolites can be uptaken by a specific cell type, experimental rates can be used to validate and constrain the model prior to the reconstruction. Sertbaş2014 and MartínJiménez2017 collected 14 and 23 flux rates corresponding to hypoxia in astrocyte and healthy astrocyte–neuron models, respectively (see Supplementary File S2 Tables S6–S8).

The second type of manual curation of brain GEMs consists of the addition of new brain-specific reactions. For example, Lewis2010 added manually reactions for the acetylcholine synthesis, which is decreased in the neurons of AD patients. These reactions were identified by flux balance analysis on the generic reconstruction Recon 1. Meanwhile, reactions linked to the ARSA gene, which is responsible for the degradation of the sulfatides in the myelin sheath, were added in EcheverriPeña2021.

### 3.12. The Completeness Is Highly Variable between the Models While Having a Similar Specificity

The size of the models in terms of the number of reactions, metabolites and genes varies greatly between the models and ranges from 639 to a median of 5942 reactions for Baloni2020 (see Table 2), and only 35 genes were shared among the models after the conversion of the model gene identifiers to ENTREZ gene identifiers (Supplementary File S1 Figure S1). This low overlap results to some extent from the comparison between bi-cellular glial-neuron, astrocyte, and whole-brain models. However, the low overlap also results from the strategy used during the model building. Two bottom-up models (Sertbaş2014 and Özcan2016) were smaller and focused mainly on the central brain metabolism. The size of the remaining seven models correlated with the size of the reconstruction used as a template for the building process that varies between 2469 consistent reactions for Recon 1 to 10,600 for Recon3D.

**Figure 2:**
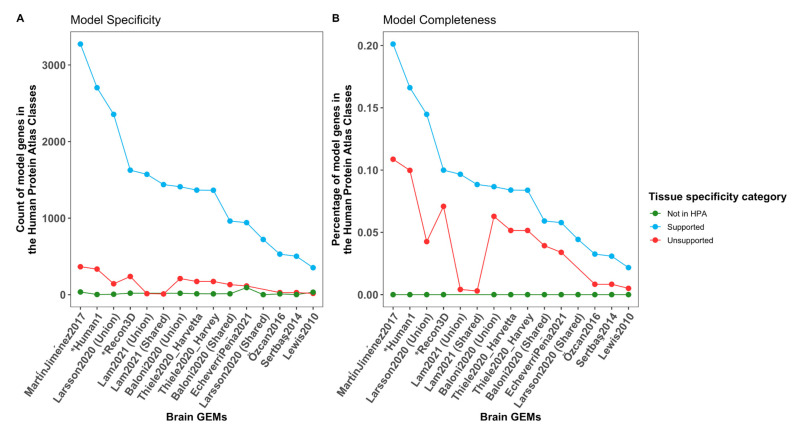
Completeness of the human brain metabolic reconstructions is linked to less specificity according to the Human Protein Atlas brain-specific category. (**A**) The genes of the brain reconstructions in addition to the Recon3D model and Human1 were classified into five categories based on differential tissue expression of the brain. These five categories were grouped into supported (in blue) and unsupported (in red). Model genes outside the HPA coding genes were coloured in blue. (**B**) Since the total number of genes in each category differs, completeness was computed as the ratio of model genes in a category and the total number of genes in that category. The number and completeness of supported and unsupported genes are higher in MartínJiménez2017 than in Human1, which indicates the loss of brain specificity by increasing the completeness of the model. Generic models are highlighted with “*”.

MartínJiménez2017 has 948 genes that were not included in any of the other brain models (Supplementary File S1 Figure S1) but also has the highest number of supported and unsupported genes by the HPA protein data in the brain according to the HPA (Figure 2). Similarly, Thiele2020 and Baloni2020 share 2762 (26.1%) and a median of 5110 (48.2%) reactions, respectively, with the Recon3D model. The ratio between the supported and unsupported genes in the brain is rather conserved across the brain models and generic GEMs (Figure 2A), showing that, to include more supported genes in the brain, inactive reactions in the brain had to be included. In terms of completeness, MartínJiménez2017 included a higher percentage of the supported and unsupported genes in the brain. Taken together, two strategies were used, bottom-up (Sertbaş2014 and Özcan2016) and top-down (MartínJiménez2017, Thiele2020, Baloni2020, EcheverriPeña2021, Lam2021, Larsson2020), that do not dictate the quality of the model but rather have an impact on their size. Lewis2010 used a compromise between the two approaches by reconstructing a subnetwork using GIMME and expression data. While focusing mainly on three pathways and the fulfilment of metabolic tasks associated with the synthesis and metabolism of acetylcholine, the inclusion of transcriptomic data allowed us to obtain a larger model than the ones using the bottom-up approach. Regarding specificity and completeness, increasing the number of brain-specific reactions causes the inclusion of genes that are considered unsupported by the HPA [90].

### 3.13. Glutamine/Glutamate/GABA Exchange Is a Brain-Specific Objective Function for Non-Glioma Models

The choice of the OF and its formulation should be tailored to the modelled cell type and condition. Thus, we compared the OFs used for non-glioma and glioma models to evaluate their relevance to brain functions (see Table 3). The OF is a reaction with the set of metabolites needed for a cell to carry out a specific task. The main task of the neuron cells is resetting the action potential by Na^+^/K^+^ ATPase, which is costly in energy [124]. This energy generated as ATP comes from either glycolysis or tricarboxylic acid cycle and OXPHOS. Many hypotheses have been proposed for the specific roles of glial and neuronal cells in the transport of energy substrates, such as the astrocyte–neuron lactate shuttle theory (ANLS) [78]. The ANLS theory states that the glucose is transported from the blood vessels to the astrocyte and then metabolised through glycolysis to produce lactate supplied to neurons. Hence, lactate production could be used as OF. However, for non-glioma, like for other healthy tissues, ATP production or maintenance is more commonly chosen. In the whole-brain Thiele2020, two maintenance OFs were used: biomass_maintenance and biomass_maintenance_noTrTr in normal and fasting conditions, respectively. In the brain bi-cellular models, glutamate, glutamine and GABA cycles are used as an additional OF to ensure a coupling between the two models. Furthermore, MartínJiménez2017 used only glutamate uptake and glutamine release for their role in the detoxification of neurotransmitters from the CSF. In summary, ATP production, biomass maintenance, glutamate, glutamine, GABA cycles and neurotransmitter exchange reactions can be used as OFs for non-glioma brain models depending on the cell type.

### 3.14. GABA and Ornithine Were Included in the Biomass Formulation of a GBM-Specific Biomass Function

**Figure 3:**
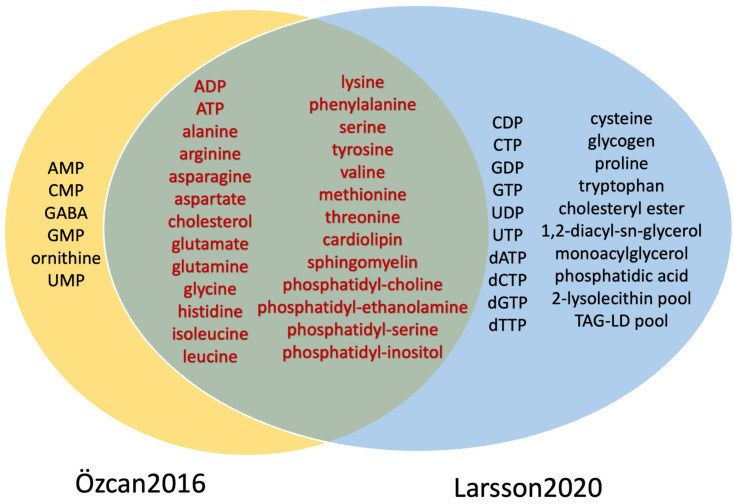
GABA, ornithine and some phospholipids are different between the tailored glioblastoma and the generic OFs. Two brain GEMs have a biomass function: Özcan2016 and Larsson2020. Both models’ OFs share 26 metabolites, mostly amino acids, cholesterol, and phospholipids. While Özcan2016’s OF has six unique metabolites, notably GABA and ornithine, Larsson2020’s OF has 20 unique metabolites such as cysteine, glycogen, proline, tryptophan, nucleotides and fatty acids.

Only Özcan2016 and Larsson2020 are modelling high-proliferative cells, and, accordingly, they used the biomass reaction as an OF. While Larsson2020 used the generic biomass function included in all HMR reconstructions, Özcan2016 built a tailored biomass function for glioma that could be adapted to future GBM models. Özcan2016 added to the healthy Sertbaş2014 24 pseudo reactions and a final biomass reaction for which the coefficients were adjusted in function of the contribution of each cell type of the white matter (94% in glial and 6% in neuron). By comparing the metabolite composition of the two OFs, we identified some differences between the two models, notably, GABA and ornithine present uniquely in Özcan2016 and, glycogen, cysteine, proline, and tryptophan (included in the generic biomass function of Larsson2020) (Figure 3). In addition, Larsson2020’s OF shows a higher diversity of phospholipids than Özcan2016, as the former is reconstructed from the generic HMR2 that covers the lipid metabolism exhaustively [107]. The neurotransmitter GABA, which is missing in the Larsson2020’s OF, was shown to control the proliferation and growth of glioma [24]. Meanwhile, glycogen, which is absent in Özcan2016’s OF, is required for cancer cell survival [125] and optimal glucose utilisation under hypoxia conditions [126]. As a result, GABA and glycogen should be potentially added to future GBM OFs.

### 3.15. CRISPR-CAS9 Screens, Experimental Fluxes and Simulating Metabolic Dysregulation Are Used as Validation

Validation of the various in silico predictions produced with the metabolic models is crucial for ensuring the quality of the curated, semi-curated or AG models.. Sertbaş2014 and Özcan2016 compared the predicted and measured flux rates for healthy and GBM, respectively. In addition, Lewis2010 validated the predicted cholinergic neurotransmission and ATP production rates with experimental data. Larsson2020 compared the predicted essential genes for GBM, against high throughput CRISPR-Cas9 data [123]. Meanwhile, MartínJiménez2017 collected the dysregulated metabolic reactions (up- or down-regulations) in metachromatic leukodystrophy from literature to compare the predicted dysregulated reactions. Flux rates can thus be employed for either model contextualisation or validation, as long as the same data are not used for both. Furthermore, in the absence of experimental data, information on the up- and down-regulation of metabolic pathways of a disease retrieved from different literature can be used as an alternative for validation.

## 4. Discussion

### 4.1. Limitations in the Brain Models Include Non-Standard Reaction Identifiers and the Use of Outdated Model-Building Algorithms

This review focused on human brain metabolic models summarising the different resources used for building better brain models. These models resembled differences in cell type from uni- and bi-cellular models, whole-brain and region-specific models. While the previous nine models gather information and data which can be employed for reconstructing future brain-specific models, the models themselves have some limitations that restrict their future use (see the summary of the strengths and drawbacks in Table 4). EcheverriPeña2021 only used the unchanged flux of neurotransmitters after ARSA knockout as a quality check and would require a more thorough validation before any future use. Because the link between TERT mutation and AD is still debated [127], using TERT mutation of zebrafish in Lam2021 as a validation of telomeric ageing in AD and PD may be insufficient. The two curated models (Sertbaş2014 and Özcan2016) use non-standard reaction identifiers, making modifications or comparisons to databases or other models more difficult [128,129]. Moreover, EcheverriPeña2021 integrated two AG models built by MinMax [103], an algorithm published in 2008 and no longer considered to conform to the state-of-the-art, from tissue-specific expression data and Recon2 [102]. While Baloni2020 was built using the Recon3D model, a heuristic threshold of the top 25-percentile was used to discretise the transcriptomic data, which strongly affects the quality of the models as shown by Opdam et al. [130]. Furthermore, unlike Thiele2020, manual curation with constraints and added reactions in Baloni2020 were applied after the building by mCADRE. This resulted in blocked reactions in Baloni2020 that were solved using 398 sink reactions. Likewise, in EcheverriPeña2021, manual curation was mostly applied to combine two AG models. Instead of using the generic model HMR3 itself, Lam2021 was built from an adipocyte-specific GEM after mapping the *gprRules* from HMR3, which may not be directly relevant to brain function. Lewis2010 was based on Recon1 (2007) [92], which has numerous shortcomings. The *metFormulas* field, which determines the chemical elements of each metabolite, was missing in four models (Sertbaş2014, Özcan2016, MartínJiménez2017, EcheverriPeña2021). This missing field prevented evaluating the mass balance of these models with MEMOTE [131]. Some brain GEMs incorporated boundary constraints from previous GEMs, without the required recalculation due to the use of different input reconstructions and biomass formulations. Despite the drawbacks of these reconstructions, the resources employed by these models can be reused (see Table 4). Finally, among the nine brain models, Thiele2020 and MartínJiménez2017 are the most curated models and, unlike Sertbaş2014 and Özcan2016, use standard annotations and are larger. Thiele2020 was built using state-of-the-art context-specific algorithms and reconstructions [88]. Furthermore, constraints and brain-specific reactions obtained from literature were fed to FASTCORE [79] already as input, allowing for building of higher quality models. MartínJiménez2017, in the pursuit of completeness, might have also lost specificity. Generally, using AG or semi-curated models with only a few refinements built by older algorithms and input reconstruction should be avoided. Instead, it would be advisable to rebuild the models using Recon3D [98] or Human1 [132] and more recently published building algorithms, while integrating the resources of the previous models as input for the algorithms (Table 4).

### 4.2. A High Completeness Is Obtained at the Cost of the Specificity

The selected models presented in this review follow two different approaches. The first is a bottom-up approach that aims to build a model around a few brain-specific pathways. The second is a top-down approach that aims to remove inactive pathways in the brain from a generic reconstruction, a database or an expression data. While bottom-up approaches were less comprehensive and often not genome-scale, the top-down strategies were lacking in the review paper in specificity, with the ratio of highly versus unsupported in brain models comparable to the generic GEM used as input. An enrichment of tissue-specific genes and reactions is expected in context-specific models compared to their input reconstruction [133]. This lack of specificity could have resulted from the choice of the low expression threshold and/or the use of data from different brain regions with different metabolisms that blurred the specificities of each area. Thus, the balance between completeness and specificity should be observed during building brain models.

### 4.3. Using Standard Identifiers and Confidence Scores Are Required for Model Comparison and Improvement

Furthermore, using non-standardised identifiers for reactions and metabolites renders the reuse of Sertbaş2014 and Özcan2016 more difficult. In general, GEMs should be built with Ensembl transcript identifiers over ENTREZ gene identifiers as different transcripts might code for different isoforms that are not all functional [128,134]. Added reactions should highlight the amount of supporting literature. They should preferably have at least two supporting publications that prove experimentally that a reaction occurs in the tissue of interest. For semi-curated and AG models, it is advisable to use the gathered information from these studies, and reconstruct new models with a state-of-the-art model-building algorithm [133] and a recent generic reconstruction such as Recon3D and Human1, rather than using the models directly. Moreover, heuristic thresholds for discretization during model-building should be avoided. These thresholds affect the quality of the output models [130], as the number of included genes, and by extension, reactions, is highly dependent on these thresholds. Confidence scores and supporting literature identifiers for manually added reactions are absent for some models. Therefore, confidence scores and supporting PubMed identifiers should be clarified and included as fields in the model file as SBML XML or MAT files. This confidence field should highlight if the manually added reactions are from literature, expression data, or for modelling purposes (i.e., gap-filling). In addition, several models could not be included in these studies, as being not available or in a non-standard format such as Excel files rendering their use more difficult.

### 4.4. The Application of Constraints to the Generic Model Prior to the Context-Specific Model Reconstruction Increases Predictability

The quality and extensiveness of the manual curation of these brain models varied strongly among the studies. Generally, the tailoring and inclusion of OF, adding core reactions from literature, and medium constraint exchange reactions to the BBB should be applied to the generic input model before the reconstruction with an algorithm and forced to be included in the output model. This tailoring might require some adjustment in the code of some algorithms but would avoid extensive post-reconstruction curation. After reconstruction, some refinement will still be required to include some reactions or pathways lacking support from the input transcriptomic and literature data. GEMs should be flux consistent or include the number of non-blocked reactions in the main text, as blocked reactions and reactions that can only carry a flux due to sink reactions would need to be removed for most modelling purposes. Reporting these blocked reactions would help any future manual curation replace these sink reactions based on recent biochemical evidence.

### 4.5. Constraining with Flux Rates Should Be Adjusted to the Generic Model

Medium constraints can either be binary, such as adding a BBB compartment or continuous such as flux rates or exo-metabolomics data. While the most updated list of metabolites that can bypass the BBB is used in Baloni2020, Thiele2020 also compiled a list that cannot pass this barrier, which can filter drugs and metabolites and predict blood biomarkers for brain diseases. Due to various diseases’ alterations in the BBB function, metabolites bypassing the BBB may need to be updated in the models according to the diseases under study by either metabolomics data of the CSF or based on literature search. For instance, metabolomics of the LGG identified dysregulated metabolites in the CSF [135] that can be used to update the healthy CSF composition from Thiele2020 for medium constraining of LGG. In GBM, tumour cells infiltrate and disrupt the BBB. Infiltrating GBM cells produce VEGF, downregulating the tight-junction proteins, and promoting angiogenesis and hypoxia [136]. Similarly, metabolomic analysis of NDD identified increased metabolites in the CSF such as kynurenine, ceramide, nitric oxide, neopterin, and other dysregulated metabolites that differ between NDDs [137]. Exo-metabolite data can be used to fine-tune medium constraining. The uptake and production rates of 213 metabolites of 60 cancer cell lines of NCI-60 [138] include two GBM and three astrocytoma cell lines. These flux rates were used to calculate the fluxes using a core cancer reconstruction from Recon 2, and the boundaries were then adjusted to Recon 2 (Zielinski et al., 2017 [139], Supplementary Data, “FBA constraints” sheet). In addition, >99% of the carbon demand of the cancer cells is met by these 23 metabolites. The calculated boundaries would need to be recalculated but could allow refining the boundaries of future models. Similarly, differences in the generic models and the units of flux rates should be considered while employing constraints from one model to another.

### 4.6. Metabolic Tasks of Brain Cell Functions Could Be Employed in Addition to Tailoring the OF

The previous brain models’ OFs are condition-specific, either for a healthy brain or glioma. Instead of applying the same OF for both neuronal and glial cells, the OF should be tailored to the cell type. In addition to neurotransmitter detoxification and ATP production, the OFs of glial cells could include lactate production and glutamate uptake. The OFs of the neurons may include the production of various neurotransmitters and the uptake of lactate, glutamine, and pyruvate [140]. Rather than using optimisation functions, defining tasks that should be fulfilled at a given flux rate would often make more sense. Additionally, enforcing the biomass maintenance, lactate secretion and others to have a non-zero baseline reaction could be used to model the low proliferation of healthy glial cells compared to gliomas. Even with the above-mentioned brain GEM, manually curated GEMs for LGG, microglia and other relevant cell types are still missing, and only an AG GEM for LGG has been built so far [68]. Microglia GEM can be built from expression data of microglia with the OFs taken from a curated macrophage GEM (ATP production, redox maintenance, NO production, production of extracellular matrix precursors, and polyamine production) [141]. Microglia GEM may then be further integrated into a multicellular GEM of GBM in order to understand cellular interactions between the microglia, astrocytes, neurons and GBM cells. Dendritic cells are another immune cell resident in the brain that increases tumour proliferation upon activation via glycolysis shift [142]. Other peripheral immune cells such as macrophages, monocytes, regulatory T cells and cytotoxic T lymphocytes penetrate the BBB after the damage of tumour growth [142,143]. Modelling these immune cellular interactions, especially the resident cells, with glioma GEM can help in understanding the metabolic modelling of the immune microenvironment. In general, generic biomass OF forces the addition of pathways that might not be active in some brain cells. Therefore, tailoring at least the metabolite composition of the biomass OF with the biochemical knowledge of the glioma would improve the predictions and, notably, the prediction of essential genes that are not predicted due to the inclusion of alternative pathways that are inactive in the brain.

### 4.7. Bulk Regional Expression Data of the Brain May Serve as an Alternative for Capturing Cellular Heterogeneity

Despite the recent developments of single-cell expression in capturing intercellular heterogeneity, robust and rigorously benchmarked tools for integrating single-cell expression into the metabolic model-building at genome-scale are non-existent for now. In the future, these tools might help in building accurate multicellular brain GEMs without the need for intensive manual curation. In addition, brain disorders being influenced by many cells of a specific region, they can also be affected by the impairment of other regions, e.g., cellular damage in NDD and conditioning in glioma extends to the nearby regions [144]. Regional expression profiling of the brain outweighs conventional bulk expression in capturing the regional vulnerability for different diseases [145]. Previous brain reconstructions tried to simulate brain heterogeneity through multicellular models (Özcan2016), independent regional brain models (Baloni2020), or multicellular, independent regional models (Lewis2010). The connection information (i.e., exchange reactions) of the different brain regions can help in building an interconnecting multi-regional model similar to multi-tissue models [97,146]. Similarly, a multi-regional model can be extended from the healthy brain to GBM. Regional expressional profiling using isolated GBM samples based on histomorphological features identified regional heterogeneity in five regions (infiltrating tumour, cellular tumour, pseudo-palisading cells around necrosis, leading-edge, and microvascular proliferation) [147]. These five regions were mapped recently to a proteomic model of three pathways (KRAS-, MYC-, and hypoxia). The KRAS-, MYC, and hypoxia pathways were identified with three main phenotypes: migration, proliferation, and altered metabolism, respectively [148]. Consequently, building a multi-regional reconstruction for GBM could identify the metabolic regional heterogeneity and vulnerability.

Taken together, the choice of the brain model depends on the focus of the study. To study the NDD, a bi-cellular model might be more suitable than a whole-brain model that would be more relevant for the interplay between different organs and the brain. The brain models Thiele2020 and MartínJiménez2017 can be further contextualised using a context-specific algorithm, expression data, and additional constraints to obtain more specific models. Finally, the data collected in these studies can be included in the reconstruction process of new models.

## Figures and Tables

**Table 1:** Curated, semi-curated and automatically generated human GEMs in the brain and their associated phenotypes. The list of metabolic models in the human brain was classified as curated, semi-curated or AG according to the level of manual curation after model-building. The detailed omic types for the “Data” column and the number of samples are summarised in Supplementary File S1 Table S2.

Model	Goal	Model Used as Template	Curation Status	Cell Type	Diseases	Data
Lewis2010 (*iNL403*) [91]	Building a curated bi-cellular human brain metabolic model to study AD	Recon 1 [92]	Curated	Astrocyte-Neuron	AD	-Human Protein Reference Database [112] -HINV [113] -HUPO brain proteome project [114] -Literature information for transport reactions between compartment -Constraints for neuron cell types. -Microarray data of AD
Sertbaş2014 (*iMS570*) [93]	Identifying biomarker metabolites for six NDD	Çakιr et al., 2007 [115]	Curated	Astrocyte- Neuron	Six NDD	-Microarray of the six NDD -Literature-derived constraints for a healthy brain
Özcan2016 *(iMS570g*) [94]	Metabolic rewiring pathways in three GBM subtypes	Sertbaş2014	Curated	Astrocyte- Neuron (glutamatergic, GABAergic, cholinergic)	Three GBM subtypes	-Curated growth objective function -Literature-derived constraints for 26 reactions for GBM -Microarray data of the three GBM cell lines
MartínJiménez2017 [95]	Building an astrocyte model reconstruction	HMA [96]	Curated	Astrocyte	Hypoxia	-Microarray data of foetal cortical astrocytes -Literature-derived constraints for healthy astrocyte exchange reactions
Thiele2020 [97]	Building sex-specific, multi-organ, whole-body model	Recon3D Model [98]	Curated	Whole-brain		-Human Proteome Map [116] - HPA [90] -CSF metabolites from Human Metabolome Database [99] and other resources. -Organ-specific reactions from literature
Baloni2020 [100]	Analysing the effect of bile acid synthesis in AD in different brain regions	Recon3D Model [98]	Semi-curated	Seven brain regions	AD	-RNA-Seq data for brain regions from post-mortem of normal and AD patients -Metabolomics of primary and secondary bile acids from the post-mortem brain samples -BBB reactions from Thiele2020 -Constraints from Lewis2010 -Human Protein Atlas
EcheverriPeña2021 (*Neuro-Glia_GEM*) [101]	Building a bi-cellular neuron-glial model to identify pathways linked to ARSA deficiency	Two tissue AG models from Recon 2 [102] (Glia: MODEL1310110064, neuron: MODEL1310110033)	Semi-curated	Neuron- Glia	Metachromatic leukodystrophy	-Reactions of the sulfatide degradation from the myelin band
Lam2021 [104]	Analysing telomeric ageing in AD and PD	iAdipocytes1850 [106] with gprRules from HMR3 [107]	Semi-curated	Whole-brain	AD, BD	-RNA-Seq of healthy brain from HPA [90] & GTEx [117] -CAGE expression of healthy brain samples from FANTOM5 [118] -RNA-Seq of AD and PD brain samples from Rajkumar dataset [119] and Zhang/Zheng dataset [120,121] -Single-cell RNA-Seq of AD and PD brain samples from ROSMAP [122] -Constraints from Baloni2020 [100]
Larsson2020 [108]	Predicting non-toxic essential genes for GBM & identifying metabolic pathways for GBM low & high overall survival	139 AG patient-derived models [109] using HMR2 generic reconstruction [107]	AG		GBM	-RNAseq of TCGA-GBM [110] -Healthy brain GEM from HMA [96] -CRISPR-Cas9 data for GBM [123]

**Table 2:** Model statistics for the brain GEMs. The curated and semi-curated models were retrieved as explained in Supplementary File S1 Table S1. For studies with more than two models (Larsson2020, Baloni2020 and Lam2021), the median sizes and range were computed. The number of reactions was determined for consistent models of these studies using FASTCC [79]. Since the models used different gene identifiers, the identifiers were mapped to ENTREZ genes.

Model	Reactions	Consistent Reactions	Metabolites	Genes	Gene Field Format	Number of ENTREZ Genes
Lewis2010	1073	727	987	403	ENTREZ Gene	403
Sertbaş2014	630	589	523	570	Gene Symbol	532
Özcan2016	659	644	548	569	ENTREZ Gene	569
MartínJiménez2017	5659	4848	5007	3765	Ensembl Gene	3674
Thiele2020_Harvey	3602	3510	2201	1836	ENTREZ Transcript	1548
Thiele2020_Harvetta	3602	3508	2203	1843	ENTREZ Transcript	1551
Baloni2020 *	5942 (5341–6328)	5327 (4870–5696)	3784 (2808–3926)	1684 (1524–1846)	ENTREZ Transcript	1409 (1292–1559)
EcheverriPeña2021	3831	3622	2473	1375	ENTREZ Transcript	1148
Lam2021 *	3283 (3274–3334)	2774 (2658–2815)	2122 (2118–2138)	1523 (1478–1572)	Ensembl Gene	1516 (1478–1572)
Larsson2020 *	3917 (2226–4877)	2951 (1382–3276)	1649 (1178–2086)	1840 (1103–2034)	Ensembl Gene	1838 (1102–2031)

* Brain GEMs with more than two models per study.

**Table 3:** Objective functions used in the brain-specific models and the rationales for using these objective functions. [m]: mitochondria, [x]: extracellular, [c]: cytosol.

Model	Objective Function(s)	Rationale for Choosing the OF
Lewis2010	ATP demand for both astrocyte and neuron cell: DM_atp(c): atp[c] + h2o[c] => adp[c] + h[c] + pi[c]	Production of the cholinergic neurotransmitter is ATP-dependent.
Sertbaş2014	1—Maximisation of the sum of glutamate/glutamine/GABA cycles. 2—Setting the value of the sum of the three-cycle fluxes to the optimal solution, then minimising the Euclidean norm of fluxes.	The 1st OF ensures compact coupling of the intercellular exchange between the astrocyte and neuron. The 2nd OF ensures fluxes with minimal utilisation of metabolic enzymes.
Özcan2016	Curated biomass growth reactions: 2.9404 protein + 0.9074 lipid_WM + 0.1091 RNA + 24 ATP => biomass + 24 ADP	Adjusting the contribution of neurons and astrocytes of macromolecules based on their percentage in the white matter, and the macromolecules composition of the white matter.
MartínJiménez2017	(A) ATP production: ADP[m] + 4 H+[c] + Pi[m] => ATP[m] + 3 H+[m] + H2O[m] (B) Glutamate uptake and glutamine release:Glutamate[x] + Glutamine[c] => Glutamate[c] + Glutamine[x]	The 1st OF ensures the consumption of different metabolites for energy production. The 2nd OF resembles the astrocyte role in detoxification of the extracellular glutamate produced by neurons, and secretion of glutamine needed by the neuron.
Thiele2020	The brain model did not have a default OF but rather the model included different OFs for different scenarios: 1—Biomass maintenance 2—Biomass maintenance with no transcription and translation	Biomass maintenance did not include DNA molecules (dgtp[n], dctp[n], datp[n], dttp[n]) as the brain cells do not replicate. The 2nd OF resembles a fasting condition.
Baloni2020	Equal to MartínJiménez2017	
EcheverriPeña2021	ATP synthesis	Modelling the highly oxidative state of the excited neuron releasing neurotransmitters
Lam2021	ATP synthesis	
Larsson2020	Growth OF of the generic reconstruction HMR2	

**Table 4:** Some advantages and drawbacks in the brain GEMs.

Model	Strengths	Drawbacks
Lewis2010	-Inclusion of a compartment for BBB (EndotheliumAndBlood) with 55 metabolites that can bypass through it (Supplementary File S2, Table S3) -Adding brain cell type-specific reactions from literature (Lewis et al., 2010 [91], Supplementary Table S1) -Comparison with experimental data of cholinergic neurotransmission rate	-The generic reconstruction used as input is outdated and has lots of short-comings
Sertbaş2014	-Constraining with literature-derived constraints. -Comparison with experimental flux ratios for healthy brain cells (Supplementary File S2, Table S6 and S7).	-Using non-standard reaction identifiers in the model -Missing *metFormula* field that prevents evaluating the stoichiometric consistency
Özcan2016	-Constraining with literature-derived constraints. -Comparison with experimental flux ratios for GBM (Supplementary File S2, Table S9).	-Using non-standard reaction identifiers in the model -Missing *metFormula* field that prevents evaluating the stoichiometric consistency
MartínJiménez2017	-Constraining with literature-derived constraints (Supplementary File S2, Table S8) -Validation with dysregulated reactions in ischemia (MartínJiménez et al., 2017 [95], Table 4)	-High rate of included genes that are unsupported in brains -The discretization method used for the expression data is not explained -Missing *metFormula* field that prevents evaluating the stoichiometric consistency
Thiele2020	-Extracting core reactions from literature and other expression data (Supplementary File S2, Table S5) -Defining permeable and impermeable metabolites across the BBB (Supplementary File S2, Table S3) -Defining CSF metabolic composition from different metabolomics data (Supplementary File S2, Table S4)	-Discretization of the Human Proteome Map using a heuristic threshold
Baloni2020	-Updating the list of Thiele2020 for metabolites passing the BBB (Supplementary File S2, Table S3) -Inclusion of constraints from Lewis2010 and OF from MartínJiménez2017	-Discretization of the expression data using a heuristic threshold -Manual curation on the AG models after model-building with mCADRE. -Gap filling with 389 sink reactions
EcheverriPeña2021	Adding reactions of myelin sheath degradation in oligodendrocyte.	-Individual AG models [102], used for integrating into a neuron-glial model, were built using the outdated MinMax algorithm -Manual curation by adding reactions after integrating the two AG models -Missing *metFormula* field that prevents evaluating the stoichiometric consistency
Lam2021		-Using an adipocyte GEM with *gprRules* of the generic HMR3 instead of using the genetic reconstruction itself
Larsson2020	-Removing essential toxic genes using predefined tasks for a healthy cell. -Validation of the predicted GBM essential genes against CRISPR-Cas9 data.	-AG reconstruction only

## Data Availability

The links of the brain metabolic models discussed in this review are available in Supplementary File S1 Table S1.

## Supplementary Materials

The following are available online at https://www.mdpi.com/article/10.3390/cells11162486/s1, Supplementary File S1 Figure S1: Low overlap between the genes included in the brain genome-scale metabolic models. To evaluate the overlap between the genes of the brain models, the intersection of these genes was counted in an UpSet plot. Brain models were retrieved as explained in Supplementary File S1, Table S1 in addition to two consistent generic models (Human1 and Recon3D). For studies that have more than two models (Baloni2020, Larsson2020 and Lam2021), the intersection and the union of all the model’s genes were appended into two gene lists. The *y*-axis represents the number of intersected genes between different sets on the *x*-axis, and the “Set Size” represents the total number of genes in that set, Supplementary File S1 Table S1: Public availability of brain genome-scale metabolic models, Supplementary File S1 Table S2: Detailed type of OMICs for the data used in the brain metabolic models and the number of samples, Supplementary File S2 Table S3: Identified metabolites bypassing the blood–brain barrier in four brain reconstructions from Thiele et al., 2020, Table EV9 and Baloni et al., 2020, Table S12, Supplementary File S2 Table S4: The metabolic composition of the cerebrospinal fluid used in Thiele2020 model from Thiele et al., 2020, Table EV8, Supplementary File S2 Table S5: Brain-specific core and absent reactions used to build Thiele2020 model with FASTCORE from Thiele et al., 2020, Table EV1, Supplementary File S2 Table S6: Experimental flux rates used in the astrocyte of Sertbaş2014 model from Sertbaş et al. 2014, Table 1, Supplementary File S2 Table S7: Experimental flux rates used in the neuron of Sertbaş2014 model from Sertbaş et al. 2014, Table 1, Supplementary File S2 Table S8: Experimental flux rates used in the MartínJiménez2017 hypoxic astrocyte model from MartínJiménez et al., 2017, Table 1, Supplementary File S2 Table S9: Constraints used in the Özcan2016 glioblastoma model from Özcan et al., 2016, Table 1. All published tables in Supplementary File S2 Tables S3–S9 are in CC BY 3.0 and 4.0. Supplementary File S2 Table S3: © 2022 Baloni, Funk, Yan, Yurkovich, Kueider-Paisley, Nho, Heinken, Jia, Mahmoudiandehkordi, Louie, et al (CC BY-NC-ND 4.0). Supplementary File S2 Tables S3–S5: © 2022 Thiele, Sahoo, Heinken, Hertel, Heirendt, Aurich, Fleming (CC BY 4.0). Supplementary File S2 Tables S6 and S7: © 2022 Sertbaş, Ülgen, Çakır (CC BY-NC-ND). Supplementary File S2 Table S8: © 2022 Martín-Jiménez, Salazar-Barreto, Barreto and González (CC BY). Supplementary File S2 Table S9: © 2022 Özcan and Çakır (CC BY).
