# Supplementary material for: Review of Current Human Genome-Scale Metabolic Models for Brain Cancer and Neurodegenerative Diseases
Source: Cells. 2022 Aug 10;11(16):2486. doi: 10.3390/cells11162486 (PMC9406599; doi:10.3390/cells11162486)
Supplement: Supplementary file 1 [file cells-11-02486-s001.zip › supplementary File 1.pdf]

Supplementary File 1:

	DOI	Code/Model
**Lewis2010** (*iNL403*)	DOI: 10.1038/nbt.1711	**Model:** Supplementary Model 1
**Sertbaş2014** (*iMS570*)	DOI: 10.1016/j.fob.2014.05.006	**Model:** http://www.systemsbiology.info.tr/downloads/iMS570_cobra_with_constraints.xml
**Özcan2016**	DOI: 10.3389/fnins.2016.00156	**Model:** Supplementary MateriaDataSheet2.ZIP (iMS570g_growth_implemented.xml)
**MartínJiménez2017**	DOI: 10.3389/fnagi.2017.00023	**Model**: https://www.ebi.ac.uk/biomodels/MODEL1608180000
**Thiele2020**	DOI: 10.15252/msb.20198982	**Model:** https://github.com/opencobra/COBRA.papers/tree/master/2020_WholeBodyModelling/SuppliedResults, OrganAtlas_Harvey.mat, OrganAtlas_Harvetta.mat
**Baloni2020**	DOI: 10.1016/j.xcrm.2020.100138	**Model and code:** https://github.com/PriceLab/Bile_acid_AD
**EcheverriPeña2021** (*Neuro-Glia_GEM*)	DOI: 10.1016/j.heliyon.2021.e07671	**Model**: https://github.com/dasalazarb/Neuro-Glia_GEM/blob/main/Neuron_Glia_GEM.xml<br>**Code**: https://github.com/dasalazarb/Neuro-Glia_GEM
**Lam2021**	DOI: 10.3390/biomedicines9101310	**Models:** https://github.com/SimonLammmm/ad-pd-retinoid/tree/main/supplementary-files/SuppFile2_iADPD<br><br>**Code:** https://github.com/SimonLammmm/ad-pd-retinoid
**Larsson2020**	DOI: 10.3389/fgene.2020.00381	**Patient-derived models:** https://www.ebi.ac.uk/biomodels/MODEL1707110000<br>**Code:** https://github.com/idalarsson/GEM-for-GBM

Table S1: Public availability of brain genome-scale metabolic models

Model	Transcriptomic	Proteomic	Metabolomic	Other data used for manual curation/ validation
**Lewis2010** (*iNL403*)	-Microarray data of AD (GSE5281, n=161)	-Human Protein Reference Database<br>-HINV<br>-HUPO brain proteome project		-Literature information for transport reactions for each compartment<br>- Constraints for neuron cell types.
**Sertbaş2014** (*iMS570*)	-Microarray data of six NDD (GSE26927, n=118)			-Literature-derived constraints for a healthy brain

**Özcan2016**	-Microarray data of three GBM cell lines (GSE13041-GPL570, n=54; GSE13041-GPL96, n=191; GSE13276, n=15)			-Curated growth objective function<br>-Literature-derived constraints for 26 reactions for GBM
**MartínJiménez2017**	-Microarray data of foetal cortical astrocytes (GSE53404, n=3)			-Literature-derived constraints for healthy astrocyte exchange reactions
**Thiele2020**		-Human Proteome Map<br>- HPA	- Human Metabolome Database and other resources.	- Organ-specific reactions from literature
**Baloni2020**	- RNA-Seq data for brain regions from post-mortem of normal and AD patients (https://www.synapse.org/#!Synapse:syn2580853/).	- HPA	- Metabolomics of primary and secondary bile acids from the post-mortem brain samples (https://www.synapse.org/#!Synapse:syn10235594, n=111)	- BBB reactions from **Thiele2020**<br>- Constraints from **Lewis2010**
**EcheverriPeña2021** (*Neuro-Glia_GEM*)				- Reactions of the sulfatide degradation from the myelin band
**Lam2021**	- RNA-Seq of healthy brain from HPA & GTEx<br>- CAGE expression of healthy brain samples from FANTOM5<br>- RNA-Seq of AD and PD brain samples from Rajkumar dataset and Zhang/Zheng dataset<br>- Single-cell RNA-Seq of AD and PD brain samples from ROSMAP			- Constraints from Baloni2020
**Larsson2020**	- RNAseq of TCGA-GBM (n=166)			- CRISPR-Cas9 data for GBM<br>- Healthy brain GEM from HMA

Table S2: Detailed type of OMICs for the data used in the brain metabolic models and the number of samples.

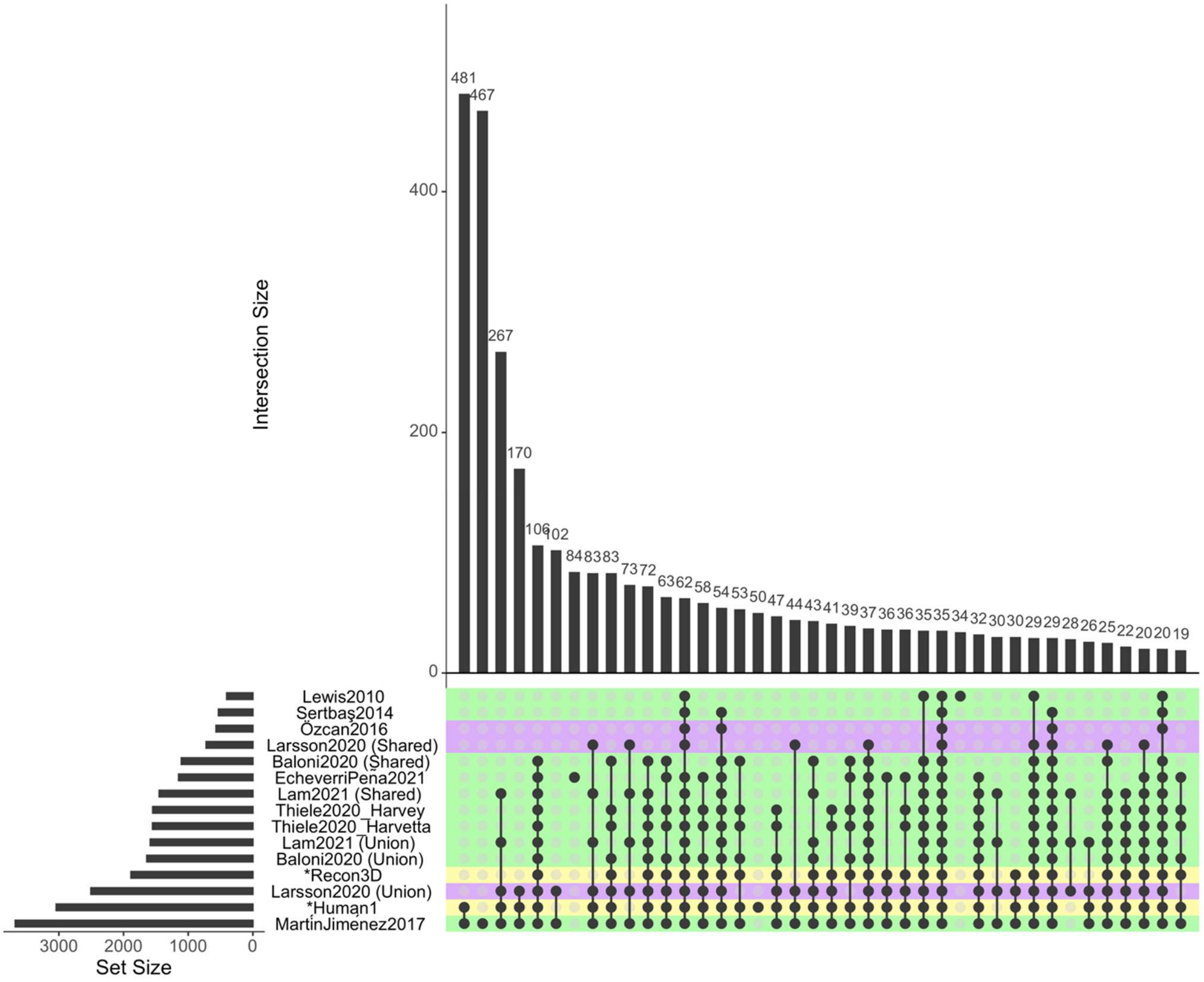

Figure S1: Low overlap between the genes included in the brain genome-scale metabolic models: To evaluate the overlap between the genes of the brain models, the intersection of these genes was counted in an UpSet plot. Brain models were retrieved as explained in **Supplementary 1, Table S1** in addition to two consistent generic models (Human1 and Recon3D). For studies that have more than two models (**Baloni2020, Larsson2020** and **Lam2021**), the intersection and the union of all the model's genes were appended into two gene lists. The y-axis represents the number of intersected genes between different sets on the x-axis, and the “Set Size" represents the total number of genes in that set.
